# Career choices for cardiology: cohort studies of UK medical graduates

**DOI:** 10.1186/1472-6920-13-10

**Published:** 2013-01-25

**Authors:** Fay Smith, Trevor W Lambert, Alex Pitcher, Michael J Goldacre

**Affiliations:** 1 UK Medical Careers Research Group, Department of Public Health, University of Oxford, Old Road Campus, Oxford OX3 7LF, UK; 2 Department of Cardiovascular Medicine, University of Oxford, John Radcliffe Hospital, Oxford, OX3 9DU, UK

**Keywords:** Cardiology, Career choice, Workforce, Medical education, Hospital medical staff

## Abstract

**Background:**

Cardiology is one of the most popular of the hospital medical specialties in the UK. It is also a highly competitive specialty in respect of the availability of higher specialty training posts. Our aims are to describe doctors’ early intentions about seeking careers in cardiology, to report on when decisions about seeking a career in cardiology are made, to compare differences between men and women doctors in the choice of cardiology, and to compare early career choices with later specialty destinations.

**Methods:**

Questionnaire surveys were sent to all UK medical graduates in selected qualification years from 1974–2009, at 1, 3, 5, 7 and 10 years after graduation.

**Results:**

One year after graduation, the percentage of doctors specifying cardiology as their first choice of long-term career rose from the mid-1990s from 2.4% (1993 cohort) to 4.2% (2005 cohort) but then fell back to 2.7% (2009 cohort). Men were more likely to give cardiology as their first choice than women (eg 4.1% of men and 1.9% of women in the 2009 cohort). The percentage of doctors who gave cardiology as their first choice of career declined between years one and five after qualification: the fall was more marked for women. 34% of respondents who specified cardiology as their sole first choice of career one year post-graduation were later working in cardiology. 24% of doctors practising as cardiologists several years after qualification had given cardiology as their sole first choice in year one. The doctors’ ‘domestic circumstances’ were a relatively unimportant influence on specialty choice for aspiring cardiologists, while ‘enthusiasm/commitment’, ‘financial prospects’, ‘experiences of the job so far’ and ‘a particular teacher/department’ were important.

**Conclusions:**

Cardiology grew as a first preference one year after graduation to 2005 but is now falling. It consistently attracts a higher percentage of men than women doctors. The correspondence between early choice and later destination was not particularly strong for cardiology, and was less strong than that for several other specialties.

## Background

Cardiology is currently a highly competitive medical specialty in the UK, attracting 6.5 applicants per higher specialty (ST3) training place in 2011 [1,2]. As with all clinical specialties, there is a need to ensure that the specialty attracts, selects and retains those junior doctors who are best suited to a future career in the field, while not raising expectations that all who want a career in cardiology will achieve one. Accordingly, it is important to understand trends in and motivations of UK medical graduates with an interest in a career in cardiology. It is also important to understand when doctors who hope to become cardiologists first make the choice; to have reliable information about the eventual career destinations of medical graduates with an initial interest in cardiology; and to understand when doctors who actually did become cardiologists first made that choice.

There are concerns regarding gender imbalance within the specialty of cardiology in the UK and internationally. According to the 2010 Royal College of Physicians’ census of consultant physicians in the UK, only 12% of consultant cardiologists (compared with 29% of all consultant physicians) and 21% of cardiology registrars (compared with 47% of all physician registrars) were women [3]. The report specifies “Cardiology remains a male-dominated specialty… The British Cardiovascular Society is attempting to address the gender imbalance by promoting the specialty to women trainees.” A survey of female specialist registrars and consultant cardiologists found that 87% would ‘recommend cardiology to a female senior house officer’, despite 43% reporting that they have experienced gender-based bias at work [4]. Gender imbalance in cardiology is also a concern in other countries [5,6]. There is a need for reliable information about the choices made for cardiology by men and women medical graduates.

The UK Medical Careers Research Group has studied the career preferences of junior doctors, across all specialties, and their subsequent career pathways, for many years. It has published the findings as they have emerged [7-10]. We have, for example, documented the fact that, at least in the past, in the UK a considerably higher percentage of men than women doctors sought careers in cardiology [11]. The aim of this paper is to summarise key findings about cardiology, across the years, and to describe doctors’ career intentions about seeking careers in cardiology, the timing of their decisions, factors that influence specialty choice, and doctors’ eventual specialty destinations. This study draws on data from twelve cohorts of doctors who graduated from UK medical schools between 1974 and 2009, reports their choices 1, 3 and 5 years after graduation, and, for doctors in the cohorts who graduated in the year 2000 or earlier, we report career destinations within and outside cardiology.

## Methods

The UK Medical Careers Research Group has surveyed the UK medical graduates of 1974, 1977, 1980, 1983, 1993, 1996, 1999, 2000, 2002, 2005, 2008 and 2009. Postal questionnaires were sent to all medical graduates from each UK medical school towards the end of the first year after graduation and at longer time intervals thereafter. Data on career choices at the end of the first year after qualification were analysed using all cohorts (1974–2009), at the third year using the 1974–2008 cohorts, and at the fifth year using the 1974–2005 cohorts (excluding the 1983s who were not surveyed at year five). In 1974 graduates of medical schools in England, Wales and Scotland were surveyed. From the 1977 cohort onwards, the surveys covered the whole of the UK including Northern Ireland. Further details of the methodology are available elsewhere [7,8]. Non-responders were sent several reminders.

The doctors were asked ‘Have you made up your mind about your choice of long-term career?’ Respondents were asked to describe their choice of specialty (or non-clinical career) in their own words, and to be as general or specific as they wished. If they had more than one specialty choice, they could enter up to three in order of preference. We also asked them to indicate whether their choices were of equal preference (which we termed ‘tied’ choices). For this paper, we excluded doctors who specified an eventual choice of career outside clinical practice and then allocated respondents to one of three groups - doctors who gave a first choice (tied or untied) for cardiology, those who gave a first choice (tied or untied) for other hospital specialties in which the consultants are physicians (except cardiology) which we term the ‘hospital physician specialties’, and those who specified a career in branches of clinical practice other than the hospital physician specialties.

Graduates from the 1993–2002 and the 2008 cohorts were further asked to indicate which factors, from a closed list of eleven factors specified in the questionnaire, had influenced their choice of specialty ‘a great deal’, ‘a little’ or ‘not at all’. Graduates from the 2009 cohort were presented with a shorter questionnaire with just four of these factors.

The data were analysed by univariate crosstabulation. To test statistical significance we used χ^2^ statistics (reporting Yates’s continuity correction where there was only one degree of freedom), and Mantel-Haenszel linear-by-linear χ^2^ test for linear association between two variables.

## Results

### Response rates

Survey questionnaires were sent to 51 323 UK doctors covering twelve cohorts (1974–2009) in the pre-registration year; 66.2% (33 974) responded. Three years after qualification the survey was sent to 44 818 doctors covering the first eleven cohorts (1974–2008); 65.6% (29 402) replied. Five years after qualification, 34 145 doctors in the 1974–2005 cohorts were surveyed, omitting the 1983 cohort; 22 602 (66.2%) responded.

### First choice of career, at years one, three and five after qualification

**Figure 1:**
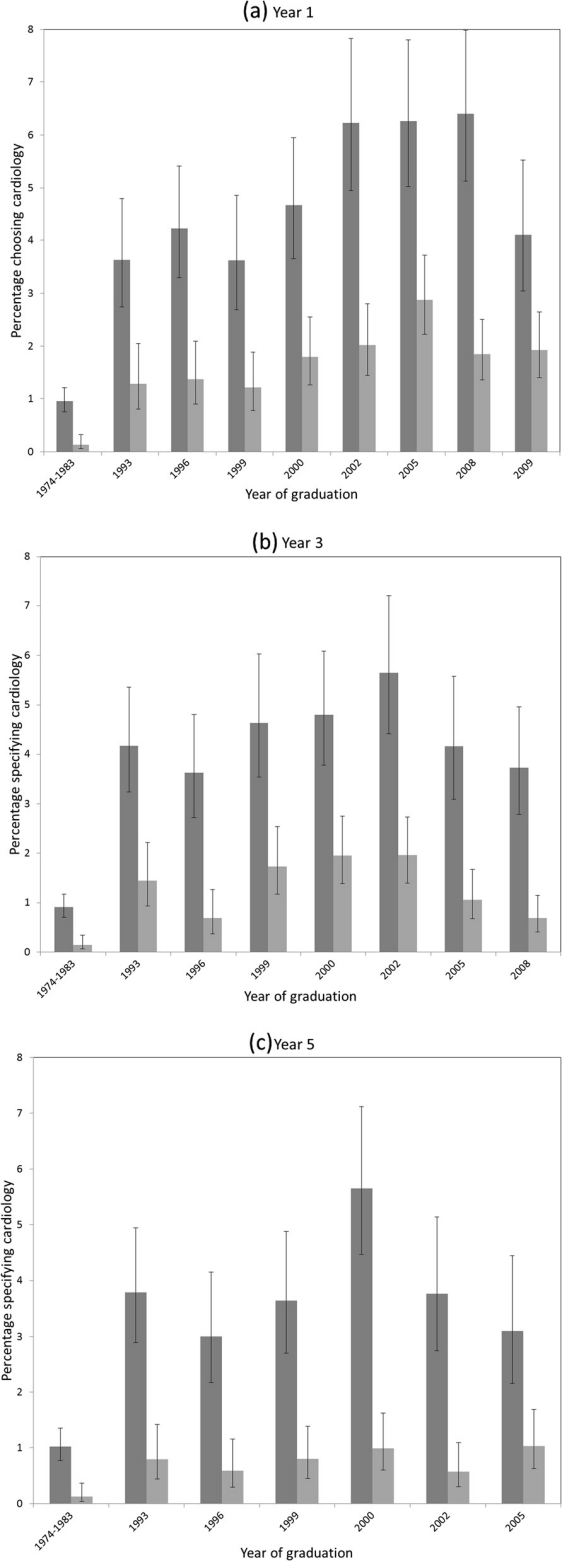
**(a)-(c): UK doctors who specified cardiology as their first choice of eventual career. **Legend: (Dark gray square-Men), (Light gray square-Women).

At year one, the percentage of doctors specifying cardiology as their first choice of long-term career rose across successive cohorts, growing from 0.7% in the 1974–1983 cohorts to 2.4% in the 1993 cohort and to a peak of 4.2% in the 2005 cohort (Figures 1a-c, Additional file 1). However, in the 2008 and 2009 cohorts the overall percentage fell to 3.4% and then to 2.7%. Doctors in the early cohorts (1974–83) were more likely to specify ‘general medicine’ than individual medical specialties within hospital medicine, which largely explains the low level of choices for cardiology in those years. Confining the analysis to doctors who qualified from 1993 to 2009, we found a significant upward linear trend over the whole period in doctors’ specifying cardiology as their career preference in year one, although the pattern was not uniform and the fall from 2005 to 2009 was substantial.

When surveyed at year three, the percentage of doctors specifying cardiology as their first choice rose from 0.6% of respondents in the 1974–1983 cohorts, to a peak of 3.4% in the 2002 cohort, but fell in the 2005 cohort to 2.2% and in the 2008 cohort to 1.8%. Five years after graduation, cardiology was the first choice of 0.7% of all respondents in the 1974–1983 cohorts, a peak of 3.0% of the 2000 cohort, and 1.8% of the 2005 cohort. There was no significant linear trend across the cohorts in year three or year five.

### Choices of men and women for cardiology

Cardiology was more popular among men than among women on all survey occasions (Figure 1a-c), but the fluctuating popularity of cardiology across cohorts applied to both men and women. Over all cohorts, in year 1, 3.2% of men and 1.5% of women made a first choice for cardiology; in year three 2.9% of men and 1.1% of women did so; and in year five the figures were 2.7% of men and 0.7% of women (Additional file 1, each comparison p < 0.001).

### Factors influencing career choice ‘a great deal’

The most important factors both for male and female doctors who specified cardiology as a first choice at all three survey times – 1, 3 and 5 years after qualification - were ‘enthusiasm/commitment’/ and ‘experience of jobs so far’ (Table 1, Additional file 2). There were few differences between men and women respondents in the rating of factors that had influenced their choices for cardiology. At year one, doctors’ concerns about the ‘hours and working conditions’ in the specialty were a less important consideration in specialty choice for men (14.4%) than for women (26.9%, χ^2^_1_ = 11.8, p < 0.01). Doctors’ considerations of their own ‘domestic circumstances’ were a more important consideration for women (19.6%) than for men (10.8%, χ^2^_1_ = 7.1, p < 0.01). ‘Financial prospects’ were a more important consideration for men (19.3%) than for women (5.3%, χ^2^_1_ = 15.0, p < 0.001). There were no statistically significant differences at year three. At year five, ‘financial prospects’ were again somewhat more important for men who had specified cardiology as a first choice (21.6%) than for women (7.7%, χ^2^_1_ = 4.4, p = 0.04).

**Table 1:** Percentages of doctors who specified each factor as influencing their choice of career ‘a great deal’

**Year 1 career choice and response**			
**Factor**	**Cardiology**	**Other hospital physician**	**All other choices**
Domestic circumstances	13.8^3**,4**^	22.6	27.0
Hours/working conditions	18.6^3**,4**^	37.5	46.5
Eventual financial prospects	14.8^1**^	9.3	13.6
Promotion/career prospects	29.3^1**,2*^	19.1	22.5
Self-appraisal	50.4	47.9	50.3
Advice from others	18.2	15.5	16.3
Student experience of subject	45.7	40.3	47.2
Particular teacher/department	39.5^1**,2**^	29.4	25.2
Inclinations before medical school	8.4^4**^	8.3	17.2
Experience of jobs so far	61.0^2**^	59.2	48.5
Enthusiasm/commitment	71.1^1*^	63.9	72.6
**Year 3 career choice and response**			
**Factor**	**Cardiology**	**Other hospital physician**	**All other choices**
Domestic circumstances	10.3^3*,4**^	20.1	27.9
Hours/working conditions	12.2^3**,4**^	36.7	54.4
Eventual financial prospects	16.0^1*^	8.5	15.9
Promotion/career prospects	24.9	20.1	25.0
Self-appraisal	51.2	48.9	51.3
Advice from others	19.7	16.4	17.0
Student experience of subject	28.5^1*^	20.2	26.4
Particular teacher/department	46.2^2**^	36.5	22.5
Inclinations before medical school	6.5^4*^	7.2	15.1
Experience of jobs so far	74.3^2*^	70.5	63.1
Enthusiasm/commitment	70.6^1*^	57.9	64.9
**Year 5 career choice and response**			
**Factor**	**Cardiology**	**Other hospital physician**	**All other choices**
Domestic circumstances	10.2^3**,4**^	31.4	44.6
Hours/working conditions	9.5^3**,4**^	44.1	61.4
Eventual financial prospects	18.9^1**,2*^	7.5	13.0
Promotion/career prospects	33.7^1**,2**^	20.6	23.8
Self-appraisal	61.9	54.8	57.4
Advice from others	19.4^2*^	15.8	13.1
Student experience of subject	29.1^1*^	20.8	23.4
Particular teacher/department	41.4^2**^	38.4	17.3
Inclinations before medical school	7.9	6.2	12.2
Experience of jobs so far	80.3^2**,1*^	71.3	63.6
Enthusiasm/commitment	88.7^1**,2**^	77.4	73.5

^1^Cardiology significantly higher percentage (p < 0.01 denoted by *, p < 0.001 denoted by **), compared with response from those who chose other hospital specialties. ^2^Cardiology significantly higher percentage, compared with response from those who chose all other specialties. ^3^Cardiology significantly lower percentage, compared with response from those who chose other hospital specialties. ^4^Cardiology significantly lower percentage, compared with responses from those who chose all other specialties.

In comparison with respondents who specified a first choice for other hospital physician specialties as an eventual career, those with first choices for cardiology attached significantly less importance (p < 0.01) to considerations of their ‘domestic circumstances’ and ‘hours and working conditions’ as factors mattering ‘a great deal’ in their choice of eventual career at years one, three, and five (Table 1). Those choosing cardiology rated ‘enthusiasm/commitment’, and ‘financial prospects’ more highly than respondents with first choices for other hospital physician specialties at all three survey times. Comparing those with first choices for cardiology with respondents who wanted careers other than in the hospital physician specialties, aspiring cardiologists rated ‘domestic circumstances’, ‘hours and working conditions’ and ‘inclinations before medical school’ lower, and they rated ‘experiences of the job so far’ and a ‘particular teacher/department’ higher.

The findings, above, are from the cohorts who graduated in 1993, 1996, 1999, 2000, 2002 and 2008. We re-examined the results by individual cohorts (adding in results from 2009, the cohort that received a briefer questionnaire) and we found very little evidence of trend across the cohorts in the importance with which the factors were regarded.

### Looking forward from early choices to later destinations

Table 2 shows early career preferences and later destinations. The results show career destinations ten years after qualification for the cohorts of 1974–1996 and seven years after for the cohorts of 1999 and 2000. 33.6% of respondents who specified cardiology as a sole first choice of long-term career one year post-graduation were later working in cardiology. Three years after qualification correspondence between choices for cardiology and destination rose to 57.8% of untied first choices for cardiology, and, five years after qualification, the great majority (87.3%) of those who indicated cardiology as their sole first choice were later practising in the specialty (Table 2). Of the doctors who specified cardiology as an early choice, men were more likely than women to be working in the specialty eventually, particularly when considering year 3 choices, where the difference reached statistical significance (χ^2^_1_ = 6.0, p = 0.014), with 42% of the women choosing cardiology in year three going on to become cardiologists, compared with 63% of the men (Table 2).

**Table 2:** Percentages of doctors who originally chose cardiology and were later working in cardiology*

	**Specialty destination: year 10 (1974–96) and year 7 (1999, 2000)**								
	**Men**			**Women**			**Total**		
Career choices	%	n	N	%	n	N	%	n	N
Year 1	35.2	38	108	28.6	10	35	33.6	48	143
Year 3	63.0	97	154	42.3	22	52	57.8	119	206
Year 5	87.3	131	150	87.1	27	31	87.3	158	181

* Ten years after qualification (1974–1996 cohorts) or seven years after (1999 and 2000 cohorts).

N denotes the number who originally chose cardiology, and n the number who later worked in it.

### Looking backward from destinations to early choices

Respondents who were practising cardiology ten (or seven) years post-graduation were grouped according to their early choices (Table 3). Of those working in cardiology, only 24% had specifically chosen cardiology in year one as their sole first choice. Over half (57%) had chosen other hospital physician specialties (including those who specified ‘general medicine’). Looking back from destinations to year three choices, 58% had made a sole first choice for cardiology and 23% had originally chosen other hospital physician specialties. About nine out of ten (88.8%) doctors in cardiology, as their career destination, had specified cardiology as their sole first choice in year five. Few practising cardiologists had specified choices outside the hospital physician specialties at years one (only 10.0%), three (3.9%) and five (1.1%). Thus, even considering year one choices, most of the movement into cardiology was by doctors who had already wanted to commit to a career in the hospital physician specialties.

**Table 3:** Percentages of practising cardiologists whose original choice was for each specified specialty group*

	**Specialty destination: year 10 (1974–96) and year 7 (1999, 2000)**					
	**Men**		**Women**		**Total**	
Career choices	%	n	%	n	%	n
**Year 1**						
Cardiology untied 1st	23.2	38	27.0	10	23.9	48
Cardiology tied 1st	3.0	5	2.7	1	3.0	6
Cardiology 2nd or 3rd	6.7	11	2.7	1	6.0	12
Any choice for other hospital physician	56.7	93	59.5	22	57.2	115
All others	10.4	17	8.1	3	10.0	20
Total	100.0	164	100.0	37	100.0	201
**Year 3**						
Cardiology untied 1st	57.7	97	61.1	22	58.3	119
Cardiology tied 1st	6.5	11	5.6	2	6.4	13
Cardiology 2nd or 3rd	9.5	16	5.6	2	8.8	18
Any choice for other hospital physician	22.0	37	25.0	9	22.5	46
All others	4.2	7	2.8	1	3.9	8
Total	100.0	168	100.0	36	100.0	204
**Year 5**						
Cardiology untied 1st	88.5	131	90.0	27	88.8	158
Cardiology tied 1st	1.4	2	0.0	0	1.1	2
Cardiology 2nd or 3rd	3.4	5	3.3	1	3.4	6
Any choice for other hospital physician	5.4	8	6.7	2	5.6	10
All others	1.4	2	0.0	0	1.1	2
Total	100.0	148	100.0	30	100.0	178

* Doctors practising cardiology at ten years after qualification (cohorts of 1974–1996) or seven years after (1999 and 2000 cohorts).

## Discussion

### Principal findings

The percentage of junior doctors selecting cardiology as their first choice of specialty, one year after qualification, increased among graduates of 1993 to 2005 and then fell among the graduates of 2008 and 2009.

We found that a progressively smaller percentage of doctors specified a career choice for cardiology beyond year one after qualification, perhaps because of difficulties in gaining places on training schemes.

Men were more likely than women to give cardiology as their first choice of future career. This difference was observed in every cohort studied, at every career time-point. Despite increased numbers of female medical graduates in the UK in recent years, cardiology is still much more attractive to men and is still one of the most male dominated specialties [3]. In addition to the differences between women and men in choices for cardiology in their early years, the attrition rate between years one and five was particularly high for women, with the gender difference most marked around year three when most doctors are starting to make their final career choices.

Considerations of working hours and the doctors’ own domestic circumstances were specified as being more important to doctors intending to pursue careers other than cardiology than they were to aspiring cardiologists. We interpret this as meaning that most aspiring cardiologists are undaunted by the rigours, in respect of possible impact on leisure hours and domestic life, of a career in cardiology.

In cardiology, early choice is not highly predictive of career destination. Only one third of doctors who specified cardiology as a sole first choice of long-term career at year one were later working in cardiology. In other specialties this ‘forward match’ rate is much higher: in the same cohorts, around 60% of doctors who specified surgery in year one, and around 80% who specified general practice in year one, eventually worked in their chosen specialty [9].

Viewed from the perspective of specialty destinations, only one quarter of doctors practising as cardiologists specified cardiology as their first choice of career in year one. This compares to ‘backward match’ rates showing that about 90% of practising surgeons, and about 60% of general practitioners, had specified their specialty as their original choice of specialty when they were in their first year after qualification [10]. It is notable, none the less, that the great majority of doctors who made a relatively late decision to commit to a career in cardiology had previously intended a career in other ‘hospital physician’ specialties including general medicine.

### Implications

A medical census in 2011 reported that 2.6% of full-time equivalent NHS medical staff in England were cardiology consultants [12]. A supply forecast in 2011 recommended no change to the numbers in training [13]. Given the high ratio of applicants per training post [1,2], and even allowing for a greater percentage of doctors working less-than-full-time in the future, it is likely that an appreciable number of aspiring cardiologists will not be able to enter the specialty. Further work is needed to find out why some doctors who initially express a first choice for cardiology subsequently stop pursuing it, and whether some of those who stop are in fact particularly well suited to a career in the specialty but are unable to obtain training posts.

The gender difference persists in choices for cardiology. As with the under-representation of women in surgery [14], it is important to ensure that there are no remediable aspects of the work or culture of the specialty that deter women who might otherwise be attracted to it.

There are a number of possible reasons for the forward and backward mismatch in cardiology choices and career destinations. These include high competition ratios, failure to gain posts, failure to progress and changes of mind. The data also reflect decisions made by those whose early choice was, say, for general medicine and who then sought a career in cardiology. As our data on the timing of specialty choice show (Tables 2 and 3), the choice of cardiology made by hospital physicians has, in the main, been successfully settled by year five.

Considerations of hours, working conditions and domestic circumstances appear, from our results, to diminish in importance as factors influencing specialty choice for cardiology (though not for other specialties) between years one and five (Table 1). One explanation for this is that some doctors who made cardiology their first choice at year one, and valued hours/working conditions, may have switched to a different career choice. It is also possible that, over time from qualification, some doctors lowered their expectations regarding hours/working conditions in line with their perceptions of the realities of the job. In this context it is notable that only 6.7% of cardiologists work less-than-whole-time compared with 16.4% for all specialties [3]. It is too soon to know whether the impact of full implementation of the European Working Time Directive may influence doctors’ specialty choice.

### Strengths and limitations

We recognise some limitations to the study. Cardiology has become increasingly sub-specialised, and it is likely that the changes in the prominence of particular sub-specialities, for example, interventional cardiology, may influence some doctors’ views about the specialty as a career choice. We did not specifically invite respondents to comment on sub-specialty choices and so were unable to address this. We survey UK medical graduates, and so cannot comment on the career choices of non-UK medical graduates who constitute an important part of the UK medical workforce. An important strength of the study is that it is based on a unique dataset, comprising multiple cohorts of medical graduates, and multiple surveys of each cohort, over 35 years. As far as we know, there are no comparable longstanding national cohort surveys like ours elsewhere. Although our study only covers the UK, we hope that it will interest others and stimulate similar work elsewhere.

## Conclusions

After growing as a first preference one year after graduation until 2005, recent data indicate that choices for cardiology are now falling. It attracts a higher percentage of men than women doctors, and steps to increase its appeal to women should be considered. It is also important to reduce the associated attrition rate among women. Many doctors choose cardiology later in their careers than is typically the case for some other specialties: it may be beneficial to doctors like these, and to the specialty of cardiology, if these doctors could be identified sooner.

### Ethical approval

This study was approved by the National Research Ethics Service, following referral to the Brighton and Mid-Sussex Research Ethics Committee in its role as a multi-centre research ethics committee (ref 04/Q1907/48).

## Competing interests

All authors have completed the Unified Competing Interest form at http://www.icmje.org/coi_disclosure.pdf (available on request from the corresponding author) and all authors want to declare: (1) financial support for the submitted work from the policy research programme, Department of Health. All authors also declare: (2) no financial relationships with commercial entities that might have an interest in the submitted work; (3) no spouses, partners, or children with relationships with commercial entities that might have an interest in the submitted work; (4) no non-financial interests that may be relevant to the submitted work.

## Authors’ contributions

MG and TL designed the study and collected the data. FS undertook the analysis, literature review and wrote the first draft. AP provided cardiology perspectives. All authors contributed to further drafts and all approve the final version.

## Authors’ information

FS: Research officer at Oxford University.

TL: Statistician at Oxford University.

AP: Clinical research fellow at Oxford University.

MG: Professor of public health at Oxford University.

## Pre-publication history

The pre-publication history for this paper can be accessed here:

http://www.biomedcentral.com/1472-6920/13/10/prepub

## Supplementary Material

Additional file 1 **Number and percentage of respondents who specified cardiology as their first choice of eventual career at one (1974–2009), three (1974–2008) and five (1974–2005) years after graduation; **Description of data: This table shows the number and percentage of doctors who specified cardiology as their first choice of long-term career (including tied choices). Click here for file

Additional file 2 **Numbers of doctors who specified each factor as influencing their choice of long-term career a great deal: one, three, and five years after graduating (1993 cohorts onwards).** Description of data: This table shows the numbers of doctors who specified each factor as influencing their choice of long-term career a great deal. Click here for file
